# Primary Care Reattendance Following an FCPP Appointment: A National Retrospective Service Evaluation

**DOI:** 10.1002/msc.70143

**Published:** 2025-06-14

**Authors:** Ben Bradford, Thomas Samuel Collier, Michael Freeman, Rob Goodwin

**Affiliations:** ^1^ Pure Unity Health Norwich UK; ^2^ School of Medicine University of Nottingham Nottingham UK

**Keywords:** delivery of health care, first contact physiotherapy practitioner, general practice, musculoskeletal conditions, primary care, service evaluation

## Abstract

**Background:**

Musculoskeletal (MSK) conditions equate to one third of a general practitioners (GP's) caseload. First contact practitioner physiotherapists (FCPPs) have been shown to be a clinically and cost‐effective alternative to GPs for managing MSK conditions. However, their impact on primary care workload(s) requires further evaluation.

**Aim:**

To determine the percentage of patients who, having seen an FCPP for a musculoskeletal disorder, then reattend with a GP, nurse practitioner (NP), or paramedic practitioner (PP) within 12 weeks, and the reasons for reattendance.

**Design and Setting:**

National, retrospective service evaluation from 70 primary care networks (PCNs) across England.

**Method:**

Data on the rate of and reasons for reattendance following an FCPP appointment were collected retrospectively between 01 January 2024 and 30 April 2024. Consent was obtained from each PCN/practice site. Extracted data consisted of patient demographics, and whether the patient reattended with a GP/NP/PP at three predetermined timepoints within 12 weeks. Reasons for reattendance at each time point were recorded against 8 predetermined criteria.

**Results:**

Two thousand one hundred forty out of 2725 patients (78.5%) did not reattend within 12 weeks of an FCPP appointment. Medication/analgesia prescribed was the most common reason for reattendance < 8 weeks and onward referral was the most common reason for reattendance between 8 and 12 weeks.

**Conclusion:**

FCPPs do have a significant impact on reducing the burden of MSK conditions in primary care. Medication was the most common reason for reattendance, supporting the advanced practice component of the FCPP role.

## 1 Introduction

General practice (otherwise known as primary care) in the United Kingdom faces an unprecedented set of challenges (Buchan et al. 2019). There are over 15.5 million people aged over 60 in the UK and the effects of an ageing population place increasing demand on the National Health Service (NHS), particularly in primary care (Greenhalgh et al. 2020). Additionally, general practice is experiencing an existential workforce crisis, driven primarily by an ever‐increasing workload and issues pertaining to recruitment and retention of general practitioners (GPs; Buchan et al. 2019; Lowe 2019). Since 2015, there has been a 2.7% decrease in the number of full‐time equivalent GPs with a disproportionate increase in demand. The size of practice populations has increased by 9% and the number of patients with chronic conditions has increased by 32%. Resultingly, over 90% of GP's report that increased workload has had a detrimental effect on their wellbeing (Gibson et al. 2017), with one third considering leaving the profession (Torjesen 2022).

Musculoskeletal (MSK) disorders account for up to 30% of the GP caseload (Morris et al. 2021). MSK conditions are estimated to cost the NHS £4.76 billion annually, and this is projected to increase further (Esbensen et al. 2020). First contact physiotherapy practitioners (FCPPs) were introduced to general practice with the creation of the additional roles reimbursement scheme (ARRS) in 2019, with the intention of bolstering the general practice workforce and in recognition of the increasing burden of MSK disorders on the health system (R. W. Goodwin and Hendrick 2016; Walsh et al. 2024). Where implemented, FCPP's perform the MSK assessment and management responsibilities historically undertaken by GPs. This extends to the diagnosis of undifferentiated presentation(s), requesting and interpreting imaging, sign‐posting patients to the most appropriate service and providing initial advice on management of MSK conditions (Greenhalgh et al. 2020; Walsh et al. 2024). FCPPs who have undergone further training are able to inject and prescribe, with the intention of further improving the efficiency of the MSK assessment and management of patients in primary care (Demont et al. 2022; Vedanayagam et al. 2021).

Since the inception of the role, there has been a small but increasing body of evidence regarding the implementation and effectiveness of FCPP's in general practice (Downie et al. 2019; Moffatt et al. 2018; Morris et al. 2021; Walsh et al. 2024; Wood et al. 2022). FCPPs offer a cost effective, safe and clinically effective alternative to a GP for the management of MSK conditions (Downie et al. 2019; Walsh et al. 2024). Patients who had initially seen an FCPP were more likely to have a statistically significant improvement in symptoms at 3 months and were less likely to incur further healthcare cost(s) over 6 months than those who had initially seen a GP (Walsh et al. 2024). In addition, high rates of patient and GP satisfaction with FCPP service(s) have been reported (Downie et al. 2019; Morris et al. 2021; Wood et al. 2022).

A potentially important measure of FCPP effectiveness is the rate at which patients reattend to see another practitioner (Stynes et al. 2021). This relatively unexplored phenomenon could indicate either a lack of FCPP clinical effectiveness in primary care or patient dissatisfaction with the FCPP support they received. Some published literature describes reattendance rates, ranging from 8.3% to 25% (R. W. Goodwin and Hendrick 2016; Ludvigsson and Enthoven 2012; Stynes et al. 2021; Walsh et al. 2024). The rate of reattendance to primary care following an FCPP appointment for each of these studies was based on relatively small sample sizes and no evidence was provided on reasons for reattendance (R. W. Goodwin and Hendrick 2016; Ludvigsson and Enthoven 2012; Stynes et al. 2021; Walsh et al. 2024). Therefore, reasons for reattendance to primary care following an FCPP appointment are currently not well understood (Moore et al. 2007).

Consequently, further work is needed to investigate whether FCPP‐led models of care have a meaningful influence on GP‐workload. One measure of assessing this could be to explore the proportion of patients who reattend with another primary care practitioner for the same MSK disorder, and to determine reasons for reattendance (Moore et al. 2007; Stynes et al. 2021; Walsh et al. 2024). The current evaluation aimed to first describe the percentage of patients who, having first seen an FCPP for a MSK disorder, then reattend with a GP, nurse practitioner (PP), or paramedic practitioner (PP) for the same issue within 12 weeks. Secondly, the evaluation sought to determine reasons for reattendance against predetermined criteria and whether reasons for reattendance vary by timepoint.

## 2 Methods

### 2.1 Aim

To determine the percentage of patients who, having first seen an FCPP for a musculoskeletal disorder, then reattend with a GP, NP or PP for the same musculoskeletal disorder within 12 weeks, and the reasons for reattendance.

### 2.2 Setting and Participants

This was a national retrospective service evaluation of adult patients (> 18 years) attending an initial FCPP appointment between 01 January 2024 and 30 April 2024. Seventy primary care networks (PCNs) distributed across six geographically determined regions in England (Table 1) were involved. FCPP team leads (FCPP‐TLs) across England were responsible for data collection. Five FCPP‐TL's involved in data collection were non‐medical prescribers. All remaining FCPP's and FCPP‐TL's were employed at equivalent to master's level agenda for change band (7) or above but had no additional non‐medical prescribing qualification(s). At each proposed PCN or GP practice site, the designated FCPP‐TL obtained consent to access medical records using a pre‐designed data sharing agreement created for the purposes of this study. FCPP‐TL's received training on the method(s) of data extraction and objectives of the evaluation. Data were extracted using a pre‐designed online data collection form. No patient identifiable data was extracted. Each FCPP‐TL was responsible for randomly evaluating the record of every other patient until 25 cases had been extracted per site (maximum of 50 cases per FCPP‐TL). Based on the workforce at the time (both FCPP's and FCPP‐TL's) it was calculated that 3475 sets of patient data for appointments conducted between the period 01 January to 30 April 2024 could be retrieved.

**TABLE 1:** Frequency of PCNs included in the data analysis across England.

Region	Number of PCNs (*N* = 70)
East Anglia	22
Northwest England	5
Midlands	9
London and Southeast England	6
South and West England	9
Yorkshire	18

### 2.3 Ethics and Reporting

As this was a service evaluation using routinely collected data, NHS REC was not required. The Health Research Authority decision tool (https://www.hra‐decisiontools.org.uk/research/redirect.html) was used and advice was sought from the partner higher education institute. The evaluation was registered with, and governance support was provided by, the organisation.

### 2.4 Data Collection—Patient Characteristics and Outcome(s)

Extracted data consisted of PCN/practice name, date of appointment, type of appointment (face‐to‐face or telephone), age and body region affected. Reattendance data included whether a patient reattended or not (Yes/No) and if so, the timepoint of their reattendance/s after an initial FCPP appointment (< 4 weeks, 4–8 weeks and 8–12 weeks) with multiple reattendances also recorded. The reason for reattendance in relation to the specific presenting MSK complaint was also recorded using predefined criteria as follows: (1) Analgesia prescribed (this comprised of any named oral, topical, transdermal or suppository medication for the purposes of pain relief); (2) Blood tests—where this was documented in the medical records and requested by the named clinician; (3) Imaging—the referral for musculoskeletal imaging (MRI, X‐ray and diagnostic ultrasound); (4) Steroid injection—the administration by sub‐cutaneous or intra‐articular means of a named corticosteroid injectate; (5) Second opinion—defined by the specific words ‘second opinion’ documented in the medical notes and/or with the specific read code ‘Further opinion sought (read code: 310343007)’ and/or ‘Patient came for second opinion (read code: 310344001)’; (6) Worsening presentation—defined by the statement of ‘*worsening*’ symptoms, pain and/or features requiring re‐assessment and/or any new or additional treatment in the patient's medical record; (7) Onward referral—referral into any secondary care service (musculoskeletal physiotherapy, hand therapy, podiatry, orthopaedics, rheumatology, pain management, triage services); (8) Med3—the issue of a fit note for modified duties or time off work.

### 2.5 Inter‐Rater Reliability

Two reviewers (BB and TC) conducted an independent review of a random sample of 100 patient records from one general practice for the purpose of determining inter‐rater reliability of the data extraction method. Inter‐rater reliability was examined across the coding of the reattendance, timeframe for reattendance and reason for reattendance variables using a method described in a previous service evaluation of patient reattendance and implications for service delivery (Moore et al. 2007). The percentage of agreement for the category reattended or not reattended was 97% for 2 raters, 100 cases and 2 categories. The percentage of agreement for the timing of reattendance was 95% for 2 raters, 100 cases and 3 categories. The percentage of agreement for the reason for reattendance was 96% for 2 raters, 100 cases and 8 categories. There was a 94% agreement for all three variables for 2 raters, 100 cases and 12 categories.

## 3 Results

### 3.1 Patient Characteristics

In total, 2725 sets of patient data were included in the final analysis. Data were collected from 70 PCNs across England (Table 1). Two thousand three hundred one appointments (84%) were face to face, and 424 were telephone/remote. The most common age‐range of patients included in the final sample was those aged 55–64 (*n* = 584), equivalent to 21%. The least common age‐range of patients was < 16 years of age (*n* = 2), equivalent to 0.07%. The most common body location of the MSK complaint was ‘lumbar spine’ (*n* = 621) equivalent to 23% and the least common was ‘non‐MSK’ (*n* = 17) equivalent to 0.6%.

### 3.2 Patient Reattendance Data

In total, 2140 out of 2725 patients (78.5%) did not reattend to see a GP, NP or PP for the same MSK issue within 12 weeks of an FCPP appointment. Of the 21.5% that did reattend, 465 (17.1%) of patients reattended on only one occasion, 99 (3.6%) reattended on two occasions and a small number (21, 0.8%) reattended on three occasions (Table 2).

**TABLE 2:** Re‐attendance rates with GP, NP or PP after an initial FCPP appointment from the total sample (*n* = 2725).

Patient reattendance	Frequency	Percentage (%)
Reattending on one occasion (*N* = 465):		
< 4 weeks	(249)	54
4–8 weeks	(135)	29
8–12 weeks	(81)	17
Reattending on two occasions (*N* = 99):		
< 4 weeks and 4–8 weeks	(52)	53
4–8 weeks and 8–12 weeks	(25)	25
< 4 weeks and 8–12 weeks	(22)	22
Reattending on all three occasions:	21	
Total	585	

### 3.3 Reasons for Re‐Attendance Data

Reasons for reattendance following an initial FCPP appointment are presented for those who reattended on a single occasion (Table 3) and those who reattended on two occasions or at all three time periods (Table 4).

**TABLE 3:** Reasons for reattendance to primary care on a single occasion.

		Category	Frequency
One occasion	0–4 weeks	1. Analgesia prescribed	82
		2. Med 3 statement	28
		3. Imaging requested	20
	4–8 weeks	1. Analgesia prescribed	30
		2. Imaging requested	21
		3. Onward referral	20
	8–12 weeks	1. Onward referral	18
		2. Analgesia prescribed	14
		3. Imaging requested	12

**TABLE 4:** Reasons for reattendance to primary care on two or more occasions.

		Most common combinations for reattendance	Frequency
Two occasions	0–4 and 4–8 weeks	1. Analgesia prescribed + analgesia prescribed.	7
		2. Med 3 statement + Med 3 statement.	4
		3. Second opinion + second opinion.	1
	0–4 and 8–12 weeks	1. Analgesia prescribed + analgesia prescribed.	6
		2. Analgesia prescribed + imaging requested.	2
		3. Med 3 statement + Med 3 statement.	2
	4–8 and 8–12 weeks	1. Analgesia prescribed + analgesia prescribed.	1
		2. Blood Investigations + blood investigations.	1
		3. Worsening presentation + worsening presentation.	1
Three occasions	0–4, 4–8 and 8–12 weeks	1. Analgesia prescribed + analgesia prescribed + analgesia prescribed.	7
		2. Analgesia prescribed + analgesia prescribed + onward referral.	2
		3. Analgesia prescribed + imaging requested + onward referral	2

17.1% of patients who attended an initial FCPP appointment reattended with a GP, NP or PP on a single occasion over the subsequent 12 weeks. If reattendance occurred within the first 4 weeks of an initial FCPP appointment, the most common reason was analgesia prescribed. The most common reason for reattendance between 4 and 8 weeks was analgesia prescribed, and onward referral for those re‐attending between 8 and 12 weeks. Of those that reattended twice during the 3‐month period (99 patients; 3.6%), the most common reason for their first reattendance was analgesia and often the second reattendance resulted in changes to, or further analgesia prescribed, or further intervention or investigation (Med 3 statement, imaging requested, blood investigations). A small number of patients (21; 0.8%) reattended three times across the 12‐week period. The most common reason(s) a patient reattended was initially for analgesia, followed by a second and third review for analgesia at subsequent time points.

## 4 Discussion

This national, retrospective service evaluation of 2725 patients demonstrated that 78.5% of patients initially seen by an FCPP do not reattend to see another member of the primary care clinical team within the subsequent 12 weeks. These findings support previous evidence that FCPPs are effective in the assessment and management of MSK conditions and in reducing the burden of MSK conditions on other members of the general practice team (R. W. Goodwin and Hendrick 2016; Ludvigsson and Enthoven 2012; Moore et al. 2007; Stynes et al. 2021; Walsh et al. 2024; Wood et al. 2022).

The most common reason(s) for a patient re‐attending following an initial FCPP appointment was for a prescription for analgesia. This was also the most common reason for repeated reattendance on two or more occasions. This could be because the initial analgesia prescribed was either ineffective or not tolerated, leading to subsequent re‐attendance. This reattendance data is informative but raises further questions. It seems to suggest that patients place particular emphasis on analgesia for the management of their MSK disorder. It might also align with evidence that suggests GPs lack confidence, and subsequently have a reliance on medication/analgesia, for the management of MSK conditions (Keavy et al. 2023; Wise et al. 2014). This finding would support the expansion of FCPP responsibilities to include prescribing as an important capability for the management of MSK disorders in primary care (Mullan et al. 2023; Noblet et al. 2022). There are currently only 2118 independent prescribing physiotherapists in the UK out of 76,920 registered physiotherapists, representing a mere 2.8% (Health Care Professions Council (HCPC) 2025; Mullan et al. 2023). However, this number is projected to increase with the government's commitment to the development of the primary care workforce as part of the NHS long term plan (Mullan et al. 2023; NHS England 2023; Noblet et al. 2022). Our findings are consistent with initiatives aimed at increasing the number of physiotherapy independent prescribers as part of a broader approach to primary care workforce transformation (Edwards et al. 2022; NHS England 2023; Noblet et al. 2022).

It is also noteworthy that other outcomes following reattendance included imaging requests, Med‐3 statement issues and onward referrals. Again, these are all elements of advanced practice extended to FCPP roles, in principle. The frequency that these activities were undertaken by other members of the general practice team suggests some inefficiencies in the MSK pathway. This also raises questions about the general practice staff understanding of the FCPP role. Previous literature has described poor GP and reception/administration staff awareness and understanding of the FCPP service and indeed this has been described as the biggest contributor to ineffective and inappropriate patient access (Greenhalgh et al. 2020; Lamb et al. 2023). Poor administration staff awareness and understanding is particularly problematic as signposting from reception has been identified as the most used route to access FCPPs (Lamb et al. 2023). Rates of reattendance could be impacted by this lack of understanding. In addition, there is evidence to suggest that patients have an entrenched perception of the GP as the default first point of contact (R. Goodwin et al. 2020) and this is even more likely if they perceive their health complaint to be serious (Morris et al. 2021). This could go some way to explain why some patients, assumingly with unresolving or worsening problems, present back to the general practice team up to three times in a 3‐month period.

### 4.1 Comparison(s) With Existing Literature

The rate of reattendance to a GP following an FCPP appointment has been reported in several other service evaluations (Downie et al. 2019; R. W. Goodwin and Hendrick 2016; Ludvigsson and Enthoven 2012; Stynes et al. 2021; Wood et al. 2022). The range of reattendance rates vary quite considerably from 8.3% to 25%, respectively (Stynes et al. 2021; Walsh et al. 2024). However, the populations in these evaluations, and study, were far smaller than the current service evaluation. Several were local evaluations that included only one or two general practices, respectively (Downie et al. 2019; Ludvigsson and Enthoven 2012). The remainder were nationwide but reported smaller numbers; Stynes et al. (2021) reported a follow‐up rate of 20% within 3 months of a consultation with an FCPP (*n* = 26/275) and Walsh et al. (2024) a follow‐up rate of 8.3% following an FCPP consultation (*n* = 23/276). In a nationwide evaluation, a success criterion of < 25% was set for patient reattendance in the 3 month following an FCPP appointment (Stynes et al. 2021). The rate of reattendance reported in the current evaluation is comfortably within the range described by other authors and under this previously described success criteria (Downie et al. 2019; NHS England and NHS Improvement 2019; Stynes et al. 2021; Walsh et al. 2024). Furthermore, this evaluation is, to the author's best knowledge, the largest retrospective service evaluation to specifically examine reattendance rates to primary care following an FCPP appointment, and the first to establish reasons for re‐attendance at three time‐points. Identifying reasons for reattendance in this manner highlights demonstratable areas for service improvement, development of the FCPP role and training or quality improvement initiatives to enhance patient and practitioner experience(s) of FCPP service(s).

A significant strength of this retrospective service evaluation is the large sample of patients included in the final analysis. The sample size of 2725 is the largest sample, to the authors’ knowledge, to retrospectively examine reattendance rates to primary care following an FCPP appointment and reasons for reattendance. This was a national sample; data were collected from 70 PCNS across England (Table 1), across a range of rural and urban locations and differing levels of social deprivation. Finally, this appears to be the first evaluation of FCPP services that has looked beyond straightforward reattendance rates in the 3 months following an FCPP appointment to provide reasons for reattendance which may aid service development or training initiatives.

This evaluation was retrospective, as such, relied on the quality of the records kept at the time by all the general practice staff. The staff at the time of consultation were unaware that the records would be accessed for the purpose of an evaluation. For example, the lumbar spine has previously been identified as the most affected joint region in primary care presentations, which is consistent with the findings from this evaluation (Jordan et al. 2010; Keavy et al. 2023; Urwin et al. 1998). Reported prevalence rates ranged from 14% to 23% (Jordan et al. 2010; Keavy 2020; Urwin et al. 1998). Reasons for inconsistent prevalence rate reporting in one study may be attributable to inaccurate diagnostic read code labels by GPs that were used to establish joint prevalence (Jordan et al. 2010). Some non‐MSK practitioners may have used a symptom code as opposed to a specific diagnostic code for knee or lumbar spine pathology. For example, low back pain because of a lumbar disc herniation causing sciatic symptoms may be given the symptom code of sciatica as opposed to lumbago with sciatica. Thus, missing the diagnostic code of lumbago for low back pain leads to a reduction in the frequency of low back pain codes, reflected in a reduced prevalence. A strength of the current study was that solely MSK specialist practitioners (FCPPs) were incorporated into the data collection, which may translate into more accurate MSK read codes, increasing the accuracy of the lumbar spine's prevalence—and that of other MSK disorders—in primary care MSK presentations. Although the data collection process showed excellent inter‐rater reliability, it was dependent on the clarity of the data recorded and this could be improved using a prospective methodology, with all stakeholders receiving advice about the evaluation and training. Even though this evaluation provides novel data about reattendance rates and the reason for reattendance, it still leaves some unanswered questions. For example, did patients reattend with another member of the general practice team due to dissatisfaction with their FCPP consultation or due to a lack of awareness of the MSK pathway constituents? Did GPs use medication/analgesia prescriptions due to patient expectations or their own lack of confidence in the management of MSK conditions? A parallel qualitative evaluation would help elucidate these questions.

### 4.2 Implications for Future Research

This evaluation is consistent with previous work in showing that reattendance rates, following an FCPP consultation, are low (approximately 20%) (R. W. Goodwin and Hendrick 2016; Ludvigsson and Enthoven 2012; Stynes et al. 2021; Walsh et al. 2024) However, a small proportion of patients (< 5%) reattend to see a GP, with the same MSK problem, several times. Although these numbers are low, they could be indicative of a lack of clarity within the broader MSK patient pathway. Some patients were seen three times within a 3‐month period. The MSK pathway, with the introduction of FCPPs and with the longer established specialist interface clinics, should negate this level of GP burden. Further research could explore this phenomenon and ascertain if it is driven by lack of patient, or GP, understanding/awareness of FCPP services or other reasons. It would also be useful to determine predictors of patient reattendance to primary care to reflect unresolved issues, pain, or dissatisfaction. For example, in the current service evaluation, analgesia was the most common reason for reattendance. However, it would be valuable to identify whether certain patient characteristics such as specific conditions (e.g., low back pain), chronicity, or psychosocial factors are associated with higher odds of reattendance. This could help refine referral pathways, enhance FCPP training, and improve the overall effectiveness of primary care musculoskeletal services by ensuring patients' needs are met more comprehensively at the first contact.

## Author Contributions

B.B., T.S.C., M.F and R.G. contributed to the conception, design and implementation of the evaluation, analysis of the results, and writing the final manuscript.

## Conflicts of Interest

The authors declare no conflicts of interest.

## Data Availability

Research data are not shared.
